# Clinical determinants of early parasitological response after ACT treatment in patients diagnosed with uncomplicated malaria in Africa: a pooled analysis of individual patient data

**DOI:** 10.1186/1475-2875-13-S1-P24

**Published:** 2014-09-22

**Authors:** Prabin Dahal

**Affiliations:** 1 WorldWide Antimalarial Resistance Network (WWARN), Oxford, UK; 2 Centre for Tropical Medicine, Nuffield Department of Clinical Medicine, University of Oxford, Oxford, UK

## Background

Artemisinin resistant *P. falciparum* malaria has emerged in Western Cambodia and now has been observed in Thailand, Vietnam and Myanmar and Lao. In addition, there is a recent report of a Vietnamese patient, apparently infected with *P. falciparum* in Angola, who was unresponsive to artemisinin treatment. This makes it even more urgent to have baseline information on early parasite response to ACTs in African patients, the first step in establishing surveillance for this important phenotype.

## Materials and methods

Individual patient data from efficacy trials conducted between 1999 and 2012 were shared with the Worldwide Antimalarial Resistance Network (WWARN) and pooled analyses conducted using standardized methodology. Factors affecting early parasitological response to treatment were investigated using logistic regression with study sites fitted as a random effect.

## Results

Data from 85 studies (N = 29,664) conducted in 27 countries with Artemether-lumefantrine (AL, n = 13,664), Artesunate-amodia-quine-fixed dose combination (ASAQ-F; n = 4,097), Artesunate-amodiaquine-co-blister combination (ASAQ-C; n = 2,505), Artesunate-amodiaquine-loose combination (ASAQ-L; n = 4,096), and Di hydro artemisinin-piperaquine (DP; n = 4,492) were included. With all drugs tested, high baseline parasite density and fever were both independent predictors of persistent parasitemia on days 1, 2 and 3. Adjusted for these variables, patients from 1 to 5 years and from 5 to 12 years in areas of low/moderate transmission were at a 2-fold and 4-fold risk of persistent parasitemia on day 3 compared to patients of the same age-group in high transmission settings. Treatment with AL was associated with a higher risk of persistent parasitemia on day 1 (AOR = 1.57 [95% CI: 1.38-1.78], *P* < 0.001) and day 2 (AOR = 1.21 [95% CI: 1.01-1.45], *P* = 0.043) compared to treatment with DP. Treatment with ASAQ-L was also associated with a higher risk of persistent parasitemia on day 2 (AOR = 1.59 [95% CI: 1.16-2.18], *P* = 0.004) and day 3 (AOR = 2.62 [95% CI: 1.24-5.53], *P* = 0.012) compared to treatment with DP.

## Conclusions

These results show no evidence of slow parasite clearance in these African studies. The greatest risk of slow clearance was observed in patients from areas of low/moderate transmission. The threshold for suspected diminished parasite susceptibility to artemisinins on day 3 was estimated to be 5%, much lower than the currently used World Health Organisation threshold of 10%. However, there are clear gaps in surveillance and any sites exceeding the day 3 parasite positivity rate of 5% should be further investigated.

